# EMBED: Essential MicroBiomE Dynamics, a dimensionality reduction approach for longitudinal microbiome studies

**DOI:** 10.1038/s41540-023-00285-6

**Published:** 2023-06-20

**Authors:** Mayar Shahin, Brian Ji, Purushottam D. Dixit

**Affiliations:** 1 Department of Physics, University of Florida, Gainesville, FL 32611 USA; 2 Physician-Scientist Training Pathway, Department of Medicine, UCSD, San Diego, CA 92103 USA; 3 Genetics Institute, University of Florida, Gainesville, FL 32611 USA; 4 Department of Chemical Engineering, University of Florida, Gainesville, FL 32611 USA; 5 Present Address: Department of Biomedical Engineering, Yale University, New Haven, CT 06511 USA

**Keywords:** Ecology, Biophysics

## Abstract

Dimensionality reduction offers unique insights into high-dimensional microbiome dynamics by leveraging collective abundance fluctuations of multiple bacteria driven by similar ecological perturbations. However, methods providing lower-dimensional representations of microbiome dynamics both at the community and individual taxa levels are not currently available. To that end, we present EMBED: **E**ssential **M**icro**B**iom**E D**ynamics, a probabilistic nonlinear tensor factorization approach. Like normal mode analysis in structural biophysics, EMBED infers ecological normal modes (ECNs), which represent the unique orthogonal modes capturing the collective behavior of microbial communities. Using multiple real and synthetic datasets, we show that a very small number of ECNs can accurately approximate microbiome dynamics. Inferred ECNs reflect specific ecological behaviors, providing natural templates along which the dynamics of individual bacteria may be partitioned. Moreover, the multi-subject treatment in EMBED systematically identifies subject-specific and universal abundance dynamics that are not detected by traditional approaches. Collectively, these results highlight the utility of EMBED as a versatile dimensionality reduction tool for studies of microbiome dynamics.

## Introduction

Advances in sequencing have enabled the characterization of host-associated microbiomes at an unprecedented resolution^1,2^. In contrast to static cross-sectional snapshots of these ecosystems, longitudinal studies offer unique insights into the biological processes structuring microbial ecosystems within individual hosts. For example, recent longitudinal studies on gut microbiome have elucidated the determinants of microbiome colonization in early childhood^3,4^, the effects of the microbiome on outcomes following bone-marrow transplant^5^, and the recolonization of microbial communities following antibiotic perturbation^6–11^.

Yet, understanding how the microbiome changes in response to environmental perturbations such as host diet variation^12,13^ and antibiotic administration^10,11^ remains challenging. This is because of the enormous organizational complexity of these ecosystems, comprising thousands of individual bacterial taxa whose abundances vary substantially across space and time^12,14–17^ and across biological replicates^18^. In addition, technical sequencing noise can seriously confound true abundance changes^15,19,20^. For example, technical noise is likely to be the most dominant factor in the observed abundance variability in more than half the bacterial taxa in longitudinal gut microbiome studies^15^ and likely remains a significant contributor for all measured taxa.

Despite this complexity, recent work suggests that abundances of individual bacterial species fluctuate with collective responses to perturbations^10–13^. Therefore, the high-dimensional dynamics of the microbiome could potentially be understood as dynamics of a few collective variables on a manifold of a much smaller dimension^21^. Indeed, approaches such as multidimensional scaling that embed microbiome samples on a smaller dimensional manifold are popular^22–24^. However, these methods only identify shifts at the community level^18^. Crucially, these methods do not account for temporal correlations in abundances of individual bacterial taxa and variability across subjects.

At the same time, there is a long history of using dimensionality reduction for multivariate time-series data^25^. Indeed, several methods have been developed in the last decade focusing specifically on the analysis of microbiome dynamics. Methods such as ecogroup identification^26^ use covariation in longitudinal data to infer interaction patterns between taxa. In contrast, methods such as MDSINE2^27^ and MTV-LMM^28^ infer interactions among species by fitting microbiome abundance dynamics to phenomenological models. Methods such as LUMINATE^20^, TGP-CODA^19^, and DIVERS^15^ quantify the magnitude of noise in abundance time series. Finally, dimensionality reduction approaches such as CTF^18^ impute lower-dimensional representations for individual subjects as well as time points using sparse tensor factorization of log-transformed data with the purpose of identifying groups of subjects with unique dynamical signatures.

In this context, we present EMBED: **E**ssential **M**icro**B**iom**E D**ynamics. EMBED is a probabilistic nonlinear tensor factorization-based dimensionality reduction method. EMBED infers common dynamical features in microbiome trajectories of multiple subjects that experience the same environmental perturbation (dietary shifts, antibiotic exposure, etc.). EMBED identifies a set of unique and orthogonal temporal bases which we call *Ecological Normal Modes* (ECNs) and taxa- and subject-specific loadings that quantify the contribution of individual ECNs in determining the abundance dynamics of taxa in individual subjects. ECNs are the statistically independent and unique dynamical templates along which the abundance trajectories of individual bacteria are decomposed. As we will show below, ECNs can also be viewed as the latent drivers of the microbial ecosystem. In systems strongly driven by environmental perturbations, they are reflective of the environmental perturbations as well as inherent dynamics of the microbiome. EMBED has several salient features. First, bacterial abundances are known to vary substantially even over short periods of time^16^. To model this variability, EMBED utilizes the exponential Gibbs–Boltzmann distribution (also known as the logistic equation). The Gibbs–Boltzmann distribution allows EMBED to capture very large changes in bacterial abundances with relatively small changes in the corresponding latents^29^. Second, by restricting the number of ECNS to be low, EMBED can provide a low-dimensional description of the community by filtering out small fluctuations in the data that may be potentially unimportant. Third, ECNs are inferred using a probabilistic model that accounts for sequencing noise inherent in all microbiome studies^15^. Fourth, similar to the normal modes in structural biology^30^, ECNs represent statistically independent modes of collective abundance changes. Fifth, the explicit multi-subject treatment in EMBED systematically identifies universal and subject-specific dynamical behaviors and bacterial taxa that exhibit that behavior.

Using synthetic data and several publicly available longitudinal datasets^12–14^, we show that EMBED-based low-dimensional approximation of microbial community dynamics is accurate and robust to sequencing noise, underscoring the low-dimensional nature of microbiome dynamics. Using synthetic data, we show that EMBED infers statistically independent dynamical modes. Using two datasets that encompass major ecological perturbations including dietary changes^13^, and antibiotic administration^10^, we show that the identified ECNs reflected specific ecological behaviors and serve as templates to reconstruct the dynamics of individual bacterial taxa. The loadings identify universal and subject-specific bacterial taxa dynamics. These results show that EMBED will be an important dimensionality reduction tool to decipher collective dynamical behaviors within the microbiome.

## Results

### EMBED identifies reduced-dimensional descriptors for longitudinal microbiome dynamics

**Fig. 1:**
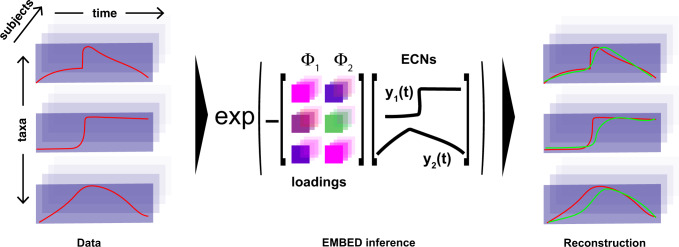
Schematic of EMBED. Dynamics of bacterial abundances within a community comprising three bacteria (left, red) is approximated using *K* = 2 ECNs $\{{y}_{k}\left(t\right)\}$ and corresponding loadings $\left\{{{\boldsymbol{\Phi }}}_{k}\right\}$ (middle). From the abundance data, EMBED identifies ECNs that are shared across subjects (right). The dynamics of abundances of individual bacteria are then approximated (green) using the inferred ECNs.

In EMBED (Fig. 1), we model microbial abundance counts ${n}_{{os}}(t)$ (Operational taxonomic unit, OTU *“o”*, subject *“s”*, and time point *“t”*) as arising from a multinomial distribution. The likelihood of observing the data is given by:

1 $$L=\prod \limits_{s,t}\frac{{N}_{s}\left(t\right)!}{\prod\limits _{o}{n}_{{os}}\left(t\right)!}\prod\limits _{o}{q}_{{os}}{\left(t\right)}^{{n}_{{os}}\left(t\right)}$$

where ${N}_{s}\left(t\right)=\sum _{o}{n}_{{os}}(t)$ is the total read count on a given day *t* for subject *s*. The probabilities ${q}_{{os}}\left(t\right)$ are modeled as a Gibbs–Boltzmann distribution^29^

2 $${q}_{{os}}\left(t\right)=\frac{1}{{\Omega }_{{st}}}{{\exp }}\left(-\mathop{\sum }\limits_{k=1}^{K}{z}_{{tk}}{\theta }_{{kos}}\right).$$

In Eq. (2), ${z}_{{tk}}$ are time-specific latents that are shared by all OTUs and subjects, ${\theta }_{{kos}}$ are OTU- and subject-specific loadings that are shared across all time points, and ${\Omega }_{{st}}$ is the normalization constant. This low-rank tensor factorization is a special case of the so-called Tucker decomposition^31^. The number of latents is chosen such that $K\ll O,T$ to obtain a reduced-dimensional description. The parameters are estimated using log-likelihood maximization. While most microbiome abundance data are compositional^32^, new techniques are being developed to measure absolute bacterial loads^15,33,34^. In addition to modeling relative abundance data, EMBED is also equipped to model measurements of absolute abundances. To do so, we use the absolute abundance instead of the daily total read count ${N}_{s}\left(t\right)$ in Eq. (1).

The optimal values of the parameters depend on the initial conditions but are nonetheless related to each other via a linear transformation^29^. We therefore identify a unique and orthonormal representation for the latents by exploiting the dynamical nature of the data. The long-term stability of the microbiome is now well-established^16,17,35^. Therefore, we fit a “return to normal” linear dynamical model to inferred latents:

3 $${{\boldsymbol{z}}}_{t+1}={\boldsymbol{A}}{{\boldsymbol{z}}}_{t}+{\boldsymbol{u}}+{\boldsymbol{\varepsilon }}{\boldsymbol{.}}$$

In Eq. (3), the matrix ***A*** is assumed to be symmetric, ***u*** are the baseline values, and the noise ***ε*** is assumed to be Gaussian distributed and uncorrelated. After diagonalizing the inferred interaction matrix (Supplementary Information section 1), ${\boldsymbol{A}}{\boldsymbol{=}}{{\boldsymbol{v}}}^{T}{\boldsymbol{\Lambda }}{\boldsymbol{v}}$, we find that the re-oriented latents, or the *ecological normal modes* (ECNs), ${{\boldsymbol{y}}}_{t}={\boldsymbol{v}}{{\boldsymbol{z}}}_{t}$ fluctuate independently of each other

4 $${y}_{t+1,k}={\Lambda }_{k}{y}_{{tk}}+{u}_{k}^{{\prime} }+{\varepsilon }_{k}^{{\prime} }.$$

In Eq. (4), ${{\boldsymbol{u}}}^{{\boldsymbol{{\prime} }}}={\boldsymbol{vu}}$, and ${{\boldsymbol{\epsilon }}}^{{\boldsymbol{{\prime}}}}={\boldsymbol{v}}{\boldsymbol{\epsilon }}{.}$ We redefine the corresponding loadings ${\boldsymbol{\Phi }}={{\boldsymbol{v}}}^{T}{\boldsymbol{\theta }}{.}$ Notably, since ${{\boldsymbol{vv}}}^{T}={\boldsymbol{I}}$, this simultaneous transformation is a mere reorientation of the latents and the loadings and does not change model predictions^29^. As we will show below, the orthonormal ECNs are uniquely defined for a given dataset. We note that the actual dynamics of the latents are likely to be more complex than the linear model (Eq. (3)). Yet, similar to normal mode analysis^30^, as we will show below, ECNs represent a reorientation of the latents that uncovers the *unique* and *orthogonal* templates of microbial abundance fluctuations.

### EMBED accurately and robustly approximates microbiome abundance time series using dynamics on a lower-dimensional manifold

Using EMBED, we approximated microbiome abundance time series from publicly available longitudinal datasets on human beings^11,12,14^ and mice^10,13^ as well as synthetic data generated using a multispecies Lotka–Volterra model^36^ (Supplementary Information section 1). When using EMBED and other reconstruction methods to model synthetic data, we sampled relative abundances using the true underlying propensities of species and a multinomial distribution with a sequencing depth of 10^4^. The accuracy of reconstruction was evaluated against the true propensities as predicted by the model. We compared EMBED with CTF (compositional tensor factorization), a recently developed dimensionality reduction method by Martino et al.^18,37^, and sparse vector autoregressive modeling (referred to as Lasso from here onwards)^38,39^. While similar to EMBED, CTF obtains both time-series reconstruction and lower-dimensional embedding, Lasso only obtains time-series reconstruction using fewer parameters than the data. To put Lasso on an equal footing with low-rank factorization methods like EMBED and CTF, the number of parameters in Lasso was adjusted to be approximately equal to EMBED and CTF by adjusting the Lagrange multiplier that dictates sparsity (# of parameters = *K* × *O* + *K* × *T* where O is the number of OTUs and T is the number of time points for a single subject time series, Supplementary Information section 2).

**Fig. 2:**
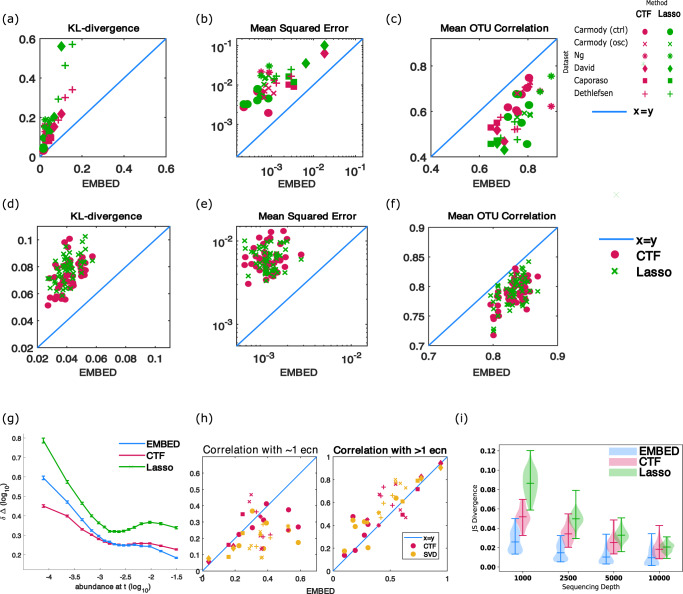
EMBED-based reconstruction of microbiome time series is accurate and precise. **a**–**f** EMBED vs CTF/Lasso reconstruction accuracy. The *x*-axis shows EMBED numbers and the *y* axis shows CTF/Lasso numbers. Colors represent different methods (green: Lasso, pink: CTF). *K* = 5 components were used in EMBED and CTF. The number of parameters in Lasso were adjusted to match the number of parameters in EMBED and CTF (see text). **a**–**c** Human and mice datasets. Individual symbols represent different datasets. **d**–**f** Synthetic data generated using the Lotka–Volterra model and sampled at a sequencing depth of 10,000. The method reconstructions are compared against the ground truth probabilities generated from the Lotka–Volterra model. **a**, **d** Kullback–Leibler (KL) divergence between the data and the reconstructed community composition. The KL divergences were normalized by sample size (number of time points). **b**, **e** Mean squared error of OTU-specific time series computed between the data and EMBED/CTF/Lasso-based reconstructions. For each time series, the error was first calculated on longitudinal trajectories of abundances of individual OTUs and then averaged over all OTUs. **c**, **f** The Pearson correlation between observed longitudinal trajectories of OTUs and the corresponding reconstruction. **g** The mean of the absolute error $\delta \Delta$ in reconstruction of OTU-specific daily abundance change $\Delta ={{{\log }}}_{10}\frac{{x}_{o}\left(t+1\right)}{{x}_{o}(t)}$ plotted as a function of OTU abundance ${x}_{o}(t)$ at time *t*. The *x* axis was binned in intervals of 5 percentiles and mean and standard errors of $\delta \Delta$ were plotted on the *y* axis. Analysis was performed by combining data across all publicly available datasets considered. **h** Fraction of taxa that correlated with only one (left) and more than one ECN (right) obtained using EMBED, temporal components obtained using CTF, and temporal component obtained using singular value decomposition of the ***zθ*** matrix. Colors represent different methods (pink: CTF, yellow: SVD). Individual symbols represent different datasets. **i** Symmetric Kullback–Leibler divergence (Jensen–Shannon divergence) between two models learned from two different multinomial samplings of the same underlying ground truth microbiome trajectories generated using the multispecies Lotka–Volterra model across different sequencing depths. The dashes represent the maximum, the mean, and the minimum of the data.

In Fig. 2, we show that EMBED-based reconstruction was significantly more accurate than CTF and Lasso both at the level of community composition as well as the dynamical trajectories of individual OTUs. Figure 2a–c show results for the publicly available datasets and Fig. 2d–f show results for the Lotka–Volterra model. Notably, as seen in Fig. 2a–f, EMBED was better at data reconstruction than CTF and Lasso for every time series. We note that the results presented below are insensitive to the dimension of the latent space (Supplementary Figs. 1 and 2) as well as the sequencing depth (Supplementary Fig. 3) and to temporally fluctuating carrying capacities in the Lotka–Volterra model (Supplementary Fig. 4). The details of the analyses can be found in Supplementary Information section 3.

Figure 2a shows the KL divergence between the observed community composition and the reconstructions based on EMBED, CTF, and Lasso. EMBED-based reconstruction was more accurate at the community level (Wilcoxon signed rank $p=1.8\times {10}^{-5}$ for the comparison between EMBED and CTF and EMBED and Lasso). Figure 2b shows that the mean squared error in OTU-specific longitudinal trajectories (averaged over OTUs) was lower in EMBED-based reconstruction (Wilcoxon signed-rank $p=1.8\times {10}^{-5}$ for the comparison between EMBED and CTF and EMBED and Lasso). Finally, in Fig. 2c, we show the Pearson correlation coefficient between the observed longitudinal time series of individual OTUs and the corresponding reconstruction. The Pearson correlation coefficient was averaged across OTUs for each subject and one number was reported per subject. This Pearson correlation coefficient was higher for EMBED (Wilcoxon signed rank $p=1.8\times {10}^{-5}$ for the comparison between EMBED and CTF and EMBED and Lasso). Figure 2d–f shows similar plots for synthetic data (Wilcoxon signed rank $p=7.5\times {10}^{-10}$ for the comparison between EMBED and CTF and EMBED and Lasso). We note that all *p-*values are identical because EMBED reconstruction was always better than CTF and Lasso reconstructions for individual datasets (not shown), leading to identical *p-*values for the nonparametric Wilcoxon test.

We next tested how the three methods perform when reconstructing OTU-specific daily abundance changes (Fig. 2g). To that end, we estimated the log ratio of daily abundance changes $\Delta ={{{\log }}}_{10}\frac{{x}_{o}(t+1)}{{x}_{o}(t)}$ across all OTUs and all days both in the publicly available time-series data and in the reconstructed time series ${\Delta }_{M}$ (M = EMBED/CTF/Lasso). We then investigated the dependence of the absolute error ${{\rm{\delta }}\Delta ={\rm{|}}\Delta -\Delta }_{M}|$ on the abundance ${x}_{o}(t)$. To that end, we binned the reconstruction error for every 5th percentile of OTU abundances ${x}_{o}(t)$. In Fig. 2g, we plot the average error for each of the 5-percentile intervals (error bars represent standard errors of the mean). Interestingly, we see that while CTF is more accurate than EMBED and Lasso at reconstructing low abundances, EMBED is more accurate in reconstructing abundance changes for highly abundant OTUs. Notably, our analysis suggests that abundance fluctuations of OTUs with mean abundance <0.1% (log_10_ = −3) are dominated by technical noise^15^. We therefore conclude that CTF-based reconstruction is accurate in modeling abundance changes that are dominated by noise, suggesting that CTF-based reconstruction may overfit to small and noise-dominated variations in OTU abundances. In contrast, EMBED-based reconstruction is more accurate compared to both CTF and Lasso for OTUs whose abundances are measured with minimal technical noise.

The reorientation *z***→***y* of latents using a dynamical model (Eqs. (3) and (4)) allows us to identify independent directions of significant collective dynamics in the microbiome without changing the accuracy of model predictions. In contrast, any other orthogonal decomposition of the microbiome time series that does not explicitly take into account dynamics is likely to result in a latent space description that involves a mixture of independent modes. To test the dynamical independence of ECNs, we used the publicly available time series as above. Each time series was approximated using EMBED using *K* = 5 ECNs. We correlated the inferred ECNs with time series of abundances of individual taxa. Correlations that were above a 5% FDR using the Benjamini–Hochberg procedure were deemed significant. As seen in Fig. 2h, on average, 35% of OTUs correlated with only one ECN while 45% of OTUs correlated with two or more ECNs. In contrast, 28% of OTUs correlated with only one component obtained using CTF (Wilcoxon signed-rank test *p*= 0.033) and 54% OTUs correlated with two or more components (Wilcoxon signed-rank test $p=0.014$). Notably, the specificity of taxon-ECN correlations was not due to the accuracy of the EMBED-based reconstruction. To test this, we performed SVD on the ***zθ*** matrix prior to the reorientation step (Eqs. (3) and (4) above) to obtain orthonormal latents ${{\boldsymbol{y}}}_{{SVD}}$ that *did not* consider the longitudinal nature of the data. We found that statistics of correlations of individual bacterial taxa with ${{\boldsymbol{y}}}_{{SVD}}$ were indistinguishable from CTF and significantly different compared to ECNs (Supplementary Table 1). These analyses underscore the importance of dynamical system-based reorientation of the latents in EMBED in identifying independent modes of significant collective abundance changes.

The probabilistic nature of EMBED accounts for spurious abundance variability arising from sampling noise. To test the robustness of EMBED to sampling noise, we generated ground truth trajectories using the multispecies Lotka–Volterra model^36^ with both competitive and cooperative interactions^40,41^. Using different sequencing depths, two sets of read counts were sampled using the same ground truth abundances. EMBED (and CTF) was used to model the observed read counts. The more robust the inference is to sampling noise, the better will be the agreement between the two inferred models. Indeed, as seen in Fig. 2i, EMBED-based reconstruction of abundance time series was internally consistent and robust to sequencing noise. The statistical significance of these results evaluated using the Wilcoxon signed-rank test can be found in Supplementary Table 2.

Based on these analyses, we conclude that EMBED can accurately and precisely reconstruct microbiome abundance time series using a small number of latent dimensions and that the inferred ECNs correspond to orthogonal modes of fluctuations in the collective dynamics of the bacterial ecosystem.

### Effect of dietary oscillations on the gut microbiome

Host diet has been shown to be a major factor influencing gut bacterial dynamics^13,42^ but in a subject-specific manner^43^. We applied EMBED to the data collected by Carmody et al.^13^ to better understand bacterial abundance changes in response to highly controlled dietary perturbations. Briefly, the diets of five individually housed mice were alternated every ∼3 days between a low-fat, plant-polysaccharide diet (LFPP) and a high-fat, high-sugar diet (HFHS). Daily fecal samples were collected for over a month (Supplementary Fig. 5).

**Fig. 3:**
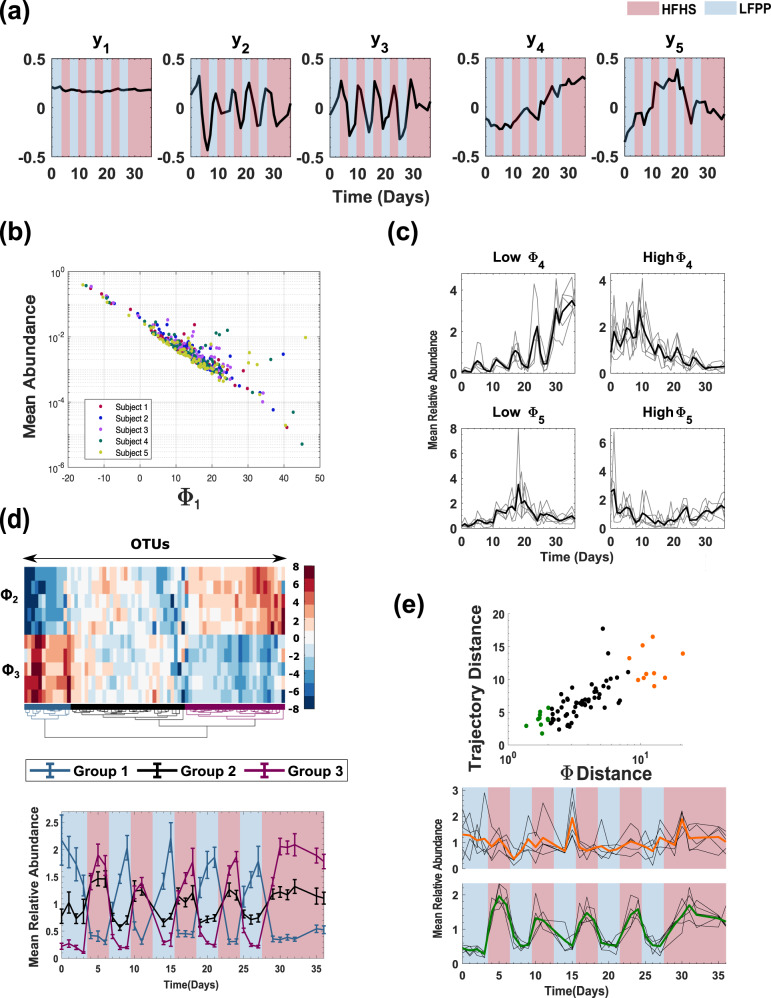
The effect of dietary oscillations on microbiome dynamics. **a** Temporal profiles of the five inferred ECNs. Blue and red panels show periods of time of administered LFPP and HFHS diets respectively. **b** The scatter plot of the feature ${{\boldsymbol{\Phi }}}_{1}$ corresponding to the first ECN and the average abundance of OTUs. **c** Top: The average abundances of five OTUs with the most negative and the most positive ${{\boldsymbol{\Phi }}}_{4}$ values. (Bottom) The average abundances of five OTUs with the most negative and the most positive ${{\boldsymbol{\Phi }}}_{5}$ values. For each subject, the abundances of the identified OTUs were first mean-normalized for each OTU, then averaged across the OTUs (faint lines). The bold lines show abundances averaged across all subjects. **d** Top: A hierarchical clustering of OTUs using the two oscillatory loadings ${{\boldsymbol{\Phi }}}_{2}$ and ${{\boldsymbol{\Phi }}}_{3}$ identifies three major groups of OTUs (colored). (Bottom) Mean relative abundance of OTUs in the three groups using the same colors as the top panel. The abundances were first mean-normalized on a per OTU basis, then averaged across subjects for each OTU, and then averaged across all OTUs in any given group. The error bars represent standard errors of mean estimated using the considered OTUs. **e** Abundance variation in top 10 OTUs that exhibit universal dynamics (green) and top 10 OTUs that show subject-specific dynamics (orange) as identified by the average subject-to-subject variability in OTU-specific **Φ** loadings.

Using *K* = 5 ECNs, EMBED obtained a lower-dimensional time-series approximation that reconstructed the original data with great accuracy (average taxa Pearson correlation coefficient $r=0.75\pm 0.18$, average community Pearson correlation coefficient, $r=0.98\pm 0.003$) (Supplementary Fig. 6). Notably, the inferred ECNs were unique (Supplementary Fig. 7), and robust to missing samples (Supplementary Fig. 8 and Supplementary Table 3) as well as variation in OTU inclusion criteria (Supplementary Fig. 9 and Supplementary Table 4). The first ECN ${y}_{1}(t)$ represented a relatively constant abundance throughout the entire time series (Fig. 3a and Supplementary Information section 3). Moreover, the corresponding loading vector ${{\boldsymbol{\Phi }}}_{1}$ showed a significant correlation to the average individual OTU abundance across time (average Spearman correlation coefficient across subjects, $r=-0.86\pm 0.06$, Fig. 2b), suggesting that despite large-scale, cyclic dietary changes, gut bacterial abundances in the community tended to fluctuate around a constant average abundance.

In contrast, ECNs ${y}_{2}(t)$ and ${y}_{3}(t)$ collectively captured the cyclic nature of dietary oscillations, confirming that the murine diet rapidly and reproducibly alters abundance dynamics even at the individual OTU level (Supplementary Information section 3). To identify OTUs whose oscillatory dynamics were similar across subjects, we clustered the loadings ${{\boldsymbol{\Phi }}}_{2}$ and ${{\boldsymbol{\Phi }}}_{3}$ of individual OTUs on ECNs ${y}_{2}(t)$ and ${y}_{3}(t)$ using Ward’s linkage. This approach is in spirit similar to clustering the log ratio of OTU dynamical trajectories reconstructed using OTU loadings corresponding only to ECNs ${y}_{2}(t)$ and ${y}_{3}(t)$ and OTU loadings corresponding only to ECN ${y}_{1}(t)$. This approach ensures that our identification of OTUs with similar dynamics is not influenced by their overall abundance. In addition to removing the effect of overall OTU abundances, EMBED also allows us to cluster OTU dynamics only along user-chosen dynamical modes. We found that bacteria in the community largely clustered into three groups (Fig. 3d); those whose abundances increased with the LFPP diet (blue, group 1), and those whose abundances increased with the HFHS diet to different extents (black and magenta, groups 2 and 3). In keeping with recent studies^44–46^, we found that the genera *Saccharicrinis*, members of the Bacteroidetes phylum, were significantly enriched in group 1 (5 out of 13 compared to 7 out of 73, hypergeometric test, $p=0.0015$) consistent with the notion that bacteria belonging to this genera are able to degrade plant polysaccharides and utilize the metabolic byproducts present in the LFPP diet.

Unexpectedly, we found two ECNs ${y}_{4}(t)$ and ${y}_{5}(t)$ that represented profound nonoscillatory behavior in abundance fluctuations. ${y}_{4}(t)$ represented an overall drift in abundance (see Supplementary Information section 3) over the time series and ${y}_{5}(t)$ represented a U-shaped recovery (see Supplementary Information section 3). The loadings corresponding to these two modes were significantly correlated across subjects (Spearman correlation coefficient $r=0.37\pm 0.16,$ averaged across mice). The top five OTUs with most negative and positive loadings ${{\boldsymbol{\Phi }}}_{4}$ (omitting OTUs that were also in the top five negative/positive for loadings ${{\boldsymbol{\Phi }}}_{5})$ experienced a significant, irreversible increase and decrease throughout the time course of the experiment respectively (Fig. 3c, top). Thus, while the dynamics of most gut bacteria in this community exhibit rapid and reversible changes in response to dietary oscillations, there exist certain bacteria that exhibit irreversible changes over time. In contrast, the top five OTUs with most negative and positive loadings ${{\boldsymbol{\Phi }}}_{5}$ (omitting OTUs that were also in the top five negative/positive for loadings ${{\boldsymbol{\Phi }}}_{4})$ experienced an inverted U-shaped and a U-shaped abundance profile (Fig. 3c, bottom). Interestingly, OTUs that exhibited these nonoscillatory behaviors differed significantly from subject to subject (Supplementary Table 5).

EMBED can identify OTUs that exhibit universal dynamics and those that exhibit subject-specific behavior. Each OTU within each subject-specific ecosystem is characterized by a *K*-dimensional vector of loadings corresponding to the *K* ECNs. OTUs whose loading vectors are similar across all subjects have similar dynamics across subjects and vice versa for OTUs with different loading vectors. To identify these universal and subject-specific OTUs, we computed the average distance across all pairs of subjects of the OTU-specific loadings vectors. This average distance also correlated strongly with the average distance of the subject-specific OTU-abundance trajectories (inset of Fig. 3e). In Fig. 3e, we plot the average abundance of ten OTUs with the most similar **Φ** loadings (bottom) and the 10 most dissimilar **Φ** loadings (top). The black lines show the OTU-averaged abundances for individual subjects and the colored bold lines (green and orange) show the average across subjects. As seen in Fig. 3e, the top ten OTUs whose dynamics were similar across all subjects strongly preferred the HFHS diet. Notably, these OTUs are overrepresented by the genus *Oscillibacter* (4 out of 10 compared to 5 out of 73, Hypergeometric test $p=9\times {10}^{-4}$). Interestingly, this overrepresentation was observed only at the genus and the family level and was *not* observed at higher taxonomic classifications (Supplementary Table 6). Moreover, no other genus or family was overrepresented. This strongly suggests a specific genus level preference to high-fat high-sugar diet in the genus *Oscillibacter* that can override subject-specific ecosystem parameters. Notably, *Oscillibacter* are known to prefer high fat^47^ as well as high-sugar diets^48^. Future work is needed to further establish the mechanistic connection between *Oscillibacter* and HFHS diets. Notably, beyond these specific associations, we found that OTU-specific dynamics across subjects was not driven by the phylogeny (Supplementary Table 7 and Supplementary Information section 4).

### ECNs identify modes of recovery of bacteria under antibiotic action

Broad-spectrum oral antibiotics have significant effects on the gut flora both during and after administration. Specifically, microbiome abundance dynamics following antibiotic administration can potentially exhibit a combination of several typical behaviors which may reflect different survival strategies^7,9,11,49^. These include quick recovery following removal of antibiotic, slow but partial recovery, and one-time changes followed by resilience to repeat antibiotic treatment. The temporal variation in abundances of any bacteria could be a combination of these typical behaviors. Moreover, given that the gut ecosystems differ across different hosts, the response of specific bacteria to the same antibiotic treatment could vary from host to host. To better parse the major modes of gut bacterial dynamics associated with antibiotic administration, we analyzed the data collected by Ng et al.^10^. Briefly, six mice were given the antibiotic ciprofloxacin in two regimens (days 1–4 and days 14–18) and fecal microbiome samples were collected daily over a period of ∼30 days (Supplementary Fig. 10).

**Fig. 4:**
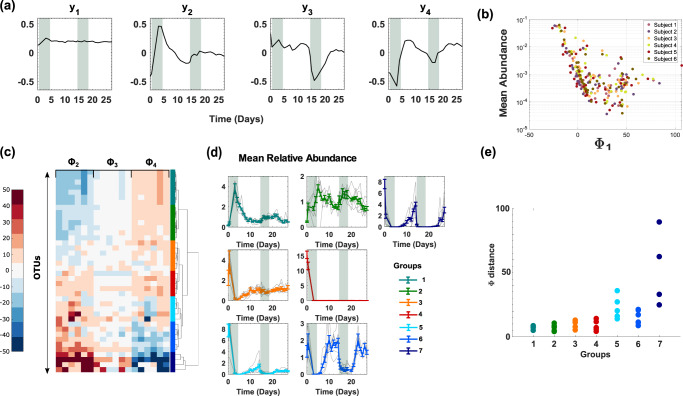
Effect of antibiotic treatment on the gut microbiome. **a** $K=4$ ECNs describe the microbiome of mice on antibiotics. The shaded region indicates the first and second doses of ciprofloxacin. **b** The scatter plot of the feature ${{\boldsymbol{\Phi }}}_{1}$ corresponding to the first ECN and the average abundance of OTUs. **c** A hierarchical clustering of OTUs using loadings except for ${{\boldsymbol{\Phi }}}_{{\boldsymbol{1}}}$. Seven major groups of OTUs with similar dynamical responses are identified from the clustering. **d** In every group and for each subject, the abundances of the identified OTUs were first mean-normalized at the OTU level. The faint lines represent subject-specific average over OTUs. The bold lines represent average across subjects. Error bars represent standard errors of mean estimated using the considered OTUs. **e** Average subject-to-subject variability in OTU-specific **Φ** loadings for the seven identified groups.

We found that a very small number *K* = 4 ECNs was sufficient to capture the data with significant accuracy (average taxa Pearson correlation coefficient $r=0.80\pm 0.2$, average community Pearson correlation coefficient, $r=0.98\pm 0.01$) (Supplementary Fig. 6). Similar to the diet study, the inferred ECNs were unique (Supplementary Fig. 7) and robust to missing samples (Supplementary Fig. 8 and Supplementary Table 3) as well as variation in OTU inclusion criteria (Supplementary Figs. 9 and Supplementary Table 4). As shown in Fig. 4a and consistent with the diet analysis, ECN ${y}_{1}(t)$ was relatively stable throughout the study (Supplementary Information section 3) and the corresponding loading vector ${{\boldsymbol{\Phi }}}_{1}$ was strongly correlated with the mean OTU abundance over time (Spearman correlation coefficient $r=-0.57\pm 0.07)$ (Fig. 4b). We found the remaining several ECNs to follow broad classes of behaviors in response to periods of stress. Indeed, ECNs, ${y}_{2}(t)$ appeared to represent an inelastic one-time change followed by a relatively stable response (Supplementary Information section 3). ECN, ${y}_{3}(t)$ represented the opposite, it responded to the antibiotic treatment the second time but not the first time. In contrast, ECN ${y}_{4}(t)$ represented *elastic* changes in the microbiome, potentially representing abundances reproducibly decreasing (or increasing) with the action of the antibiotic but quickly bouncing back to pre-antibiotic levels when it was withdrawn (Supplementary Information section 3).

These salient dynamical features were captured when we clustered the OTUs using the loadings ${{\boldsymbol{\Phi }}}_{2}-{{\boldsymbol{\Phi }}}_{4}$ using Ward’s linkage (Fig. 4c), which identified seven major groups of OTUs with distinct dynamical behaviors (Fig. 4c, d). Interestingly, while some of the groups simply reflected behaviors of individual ECNs, others could be understood according to their relative contributions across multiple ECNs. For example, the behavior of OTUs in groups 1 and 3 aligned with ECN ${y}_{2}(t)$, albeit with opposing trends. Group 1 OTUs flourished during the first antibiotic treatment but the second treatment did not elicit a similar response. In contrast, OTUs in group 3 diminished in their abundance after the first antibiotic treatment but were resistant to subsequent antibiotic action.

OTUs in groups 2, 5, 6, and 7 displayed highly elastic dynamics in response to both periods of antibiotic administration. Group 2 OTUs was overrepresented by the genus *Akkermansia* (all 2 out of 41 OTUs are in Group 2, Hypergeometric test $p=0.026$) flourished during the antibiotic treatment but decreased their abundance in a reversible manner when antibiotics were withdrawn. Notably, species from this genus are known to be rare in the human gut but only colonize it following treatment with broad-spectrum antibiotics, including ciprofloxacin^50^. OTUs in groups 5, 6, and 7 in contrast diminished their abundance in the presence of antibiotics in a reversible manner. Group 6 was overrepresented by the genus *Blautia* (3 out of 6 compared to 5 out of 41, Hypergeometric test *P* = 0.017), while group 7 was overrepresented by the genus *Aestuariispira* (all 2 out of 41 OTUs are in Group 7, Hypergeometric test *p* = 0.0073). Finally, group 4 comprised OTUs that were exquisitely sensitive to initial antibiotic administration, whose abundance did not make any meaningful recovery. These OTUs were overrepresented in the genus *Coprobacter* (2 out of 5 compared to 3 out of 41, Hypergeometric test *p* = 0.035). These specific associations need to be further investigated.

Notably, OTUs in groups 5 and 7, groups that represent slower and partial recovery compared to OTUs group 6, exhibited significant subject-to-subject variability as quantified by both the average subject-to-subject variability in OTU-specific **Φ** loadings (Fig. 4e) and the subject-to-subject variability in OTU-specific abundance trajectories (Supplementary Fig. 10). While these OTUs exhibited qualitative dynamics of recovery across all subjects (Supplementary Fig. 10), the time course and the extent of recovery varied from subject-to-subject. These findings are corroborated by recent studies that show imperfect and subject-specific recovery of bacterial abundances following antibiotic treatment^11,51–53^. Interestingly, unlike the diet study, the OTUs in the same dynamical group shared phylogenetic similarity (Supplementary Table 7 and Supplementary Information section 3).

## Discussion

Bacteria in host-associated microbiomes live in complex ecological communities governed by competitive and cooperative interactions, and a constantly changing environment. Extensive spatial and temporal variability and coordinate changes in abundances in response to environmental perturbations are a hallmark of these communities. Dimensionality reduction can leverage these fluctuations, but its use towards understanding microbiome dynamics has thus far been limited.

In this work, we presented EMBED, a dimensionality reduction approach specifically tailored to identify the *ecological normal modes* in the dynamics of bacterial communities that are shared across subjects undergoing identical environmental perturbations. Identified ECNs shed insight into the underlying structure of bacterial community dynamics. By applying EMBED to several times series datasets representing major ecological perturbations, we identified immediate and reversible changes to the gut community in response to these stimuli. However, EMBED also identified more subtle, longer-term, and perhaps irreversible changes to specific members of the community, the mechanisms, and consequences of which would be interesting to pursue further. Notably, while EMBED can learn accurate lower-dimensional representation in any longitudinal data (Supplementary Fig. 11), the inferred ECNs are likely to be easily interpretable when individual hosts are experiencing the same environmental perturbations.

One of the ECNs in the studied datasets (Figs. 3 and 4) was consistently found to be constant over time. This ECN also reflected the temporal mean abundance of individual OTUs. We can potentially leverage this insight and absorb this ECN in the lower-dimensional model. Specifically, we can model the departure from the mean abundance as a Gibbs–Boltzmann distribution. That is, instead of Eq. (1), we can model OTU abundances as

5 $${q}_{{os}}\left(t\right)=\frac{{\mu }_{{os}}}{{\Omega }_{{st}}}{{\exp }}\left(-\mathop{\sum }\limits_{k=1}^{K}{z}_{{tk}}{\theta }_{{kos}}\right).$$

where ${\mu }_{{os}}$ is the temporal average abundance of OTU “o” in subject “s”. This way, we model only the fluctuations around the mean abundance and potentially reduce the dimensionality of our description even further. We leave this for future studies.

One key parameter in EMBED is the number of components *K*. A large *K* will necessarily fit the data better, potentially fitting to noise and unimportant idiosyncrasies in the data. How do we decide the appropriate number of components? In this work, we chose *K* based on the qualitative elbow method^54^ (Supplementary Fig. 12). However, going forward, more rigorous approaches can be implemented. EMBED is a probabilistic model and information-theoretic criteria^55^ could be used to identify the correct number of components. These criteria seek a balance between an increase in the number of parameters and the accuracy of fit to data (likelihood). We note that the total likelihood of the data in our model is linearly proportional to the sequencing depth. However, the reported sequencing depth is typically over-inflated compared to the true nucleotide capture probability of the experiments leading to an inflated estimate of the total likelihood. This issue has been well discussed in single-cell RNA sequencing (see e.g.,^56^). One approach to solve this in the context of the microbiome is to obtain technical repeats^15^ which can in turn allow us to estimate the true technical noise.

The presented formulation of EMBED specifically focused on identifying dynamical features of the microbiome in hosts that were subjected to the same strong environmental perturbation. However, in many cases, the perturbations may be weak, for example, a gradual shift in diet^57^, or completely absent, for example, when studying maturation of gut microbiomes of infants^58^. In such cases, we expect a significantly higher host-to-host variability in microbiome dynamics. In this case, EMBED can be reformulated to capture this variability. Here, instead of the tensor decomposition in Eq. (2), we can model the microbiome dynamics using a tensor decomposition as follows:

6 $${q}_{{os}}\left(t\right)=\frac{1}{{\Omega }_{{st}}}{{\exp }}\left(-\mathop{\sum }\limits_{k=1}^{K}{z}_{{tk}}{\theta }_{{ok}}{\Gamma }_{{sk}}\right).$$

In Eq. (6), ${z}_{{tk}}$ are time-specific embeddings, ${\theta }_{{ok}}$ are species-specific embeddings, and ${\Gamma }_{{sk}}$ couple these embeddings to specific subjects. We leave this generalization to future studies.

While EMBED was specifically developed to study microbiomes, it reflects a more generalizable framework that can easily be applied to other types of longitudinal sequencing data as well. We therefore expect that EMBED will be a significant tool in the analysis of dynamics of high-dimensional sequencing data beyond the microbiome.

## Methods

### Inference of ECNs from longitudinal data

We consider that abundance of *O* bacterial operational taxonomic units (OTUs) are measured over a period of *T* days in *S* subjects. We model the read counts ${n}_{{os}}(t)$ of OTUs *“o”* on any given day *t* in subject *s* as a multinomial distribution. The likelihood of observing the data is given by

7 $$L=\prod\limits _{s,t}\frac{{N}_{s}\left(t\right)!}{\prod\limits _{o}{n}_{{os}}\left(t\right)!}\prod\limits _{o}{q}_{{os}}{\left(t\right)}^{{n}_{{os}}\left(t\right)}$$

where ${N}_{s}\left(t\right)=\sum _{o}{n}_{{os}}(t)$ is the total read count on a given day and ${q}_{{os}}\left(t\right)$ are the underlying propensities for individual OTUs. We model these propensities using the exponential Gibbs–Boltzmann distribution which allows us to capture large variations in OTU abundances^29^.

8 $${q}_{{os}}\left(t\right)=\frac{1}{{\Omega }_{{st}}}{{\exp }}\left(-\mathop{\sum }\limits_{k=1}^{K}{z}_{{tk}}{\theta }_{{kos}}\right)$$

where ${z}_{{tk}}$ are time-specific latents that are shared by all OTUs and subjects, and ${\theta }_{{kos}}$ are OTU-and subject-specific loadings that are shared across all time points. The number *K* of latents/loadings is chosen such that $K\ll O,T$ thereby achieving a lower-dimensional description of the time-series data. We obtain the *z*s and the *θ*s using the maximum likelihood approach. While most microbiome abundance data are compositional, new techniques are being developed to measure absolute bacterial loads^15^. EMBED is naturally equipped to model measurements of abundances. To do so, we use the absolute abundance instead of the daily total read count ${N}_{s}\left(t\right)$ in Eq. (1) (Supplementary Fig. 13).

To that end, we write down the log-likelihood of the data:

9 $${ln}={const}.+\sum\limits _{t,o,s}{n}_{{os}}\left(t\right){\rm{log }}\,{q}_{{os}}(t).$$

The constant term of the likelihood does not depend on the parameters and can thus be omitted in likelihood maximization. Simplifying using Eqs. (7) and (8), we have

10 $${ln}=-\sum\limits _{t,o,s,k}{{N}_{s}\left(t\right)x}_{{os}}\left(t\right){z}_{{tk}}{\theta }_{{kos}}-\sum\limits _{t,s}{\rm{log }}{\Omega }_{{st}}.$$

Here ${x}_{{os}}\left(t\right)={n}_{{os}}(t)/{N}_{s}(t)$ is the relative abundance of OTU *o* at time *t*. We obtain the gradients

11 $$\frac{\partial \mathrm{ln}}{\partial {z}_{{tk}}}=-\sum\limits _{o,s}{N}_{s}(t)\left({x}_{{os}}\left(t\right)-{q}_{{os}}\left(t\right)\right){\theta }_{{kos}}\,{\rm{and}}$$

12 $$\frac{\partial \mathrm{ln}}{\partial {\theta }_{{kos}}}=-\sum\limits _{t}{N}_{s}\left(t\right){z}_{{tk}}\left({x}_{{os}}\left(t\right)-{q}_{{os}}\left(t\right)\right)$$

We use gradient ascent algorithm to find the latents and the loadings that maximize the likelihood. In the analyzed datasets, the read counts on all days were equal. Therefore, we performed gradient ascent by normalizing the log-likelihood by the total read count and using relative abundances on the left-hand side of Eqs. (11) and (12). A learning rate of $\eta \in [\mathrm{0.001,0.005}]$ ensured that the inference was stable. When investigating the accuracy of EMBED-based reconstruction of community composition (Fig. 2), we stopped the inference when the relative gradients of both *z*s and *θ*s were less than ${10}^{-3}$ or if the maximum number of iterations exceeded ${10}^{5}$. When analyzing the diet and the antibiotics datasets, we stopped the inference when the relative gradients of both *z*s and *θ*s were less than ${10}^{-4}$ or if the maximum number of iterations exceeded ${10}^{6}$.

For a given *K*, using the microbiome data ${x}_{{os}}\left(t\right)$ and starting from random initialization, we first simultaneously infer the latents ${z}_{{tk}}$ and the features ${\Theta }_{{kos}}.$ We observe that the $T\times K$ matrix ***z*** of latents can be multiplied by an invertible matrix ***B*** $\left({\boldsymbol{z}}{\boldsymbol{\to }}{\boldsymbol{zB}}\right)$ and the corresponding matrix $K\times O\times S$ matrix of features can be multiplied by the inverse ${{\boldsymbol{B}}}^{{\boldsymbol{-}}{\boldsymbol{1}}}$ $\left({\boldsymbol{\Theta }}{\boldsymbol{\to }}{{\boldsymbol{B}}}^{{\boldsymbol{-}}{\boldsymbol{1}}}{\boldsymbol{\Theta }}\right)$ and the abundance predictions from the model do not change. Therefore, we use the Gram–Schmidt procedure to orthogonalize the matrix of latents such that ${\boldsymbol{z}}{\boldsymbol{\to }}{{\boldsymbol{z}}}^{{\boldsymbol{{\prime} }}}$ where ${{\boldsymbol{z}}^{\prime}}^{T}{{\boldsymbol{z}}}^{\prime}\,{\boldsymbol{=}}\,{{\boldsymbol{I}}}_{{\boldsymbol{K}}}$ is an identity matrix. For an inferred matrix of latents ***z***, we found out the matrix multiplier ${\boldsymbol{B}}\,{\boldsymbol{=}}\,{{\boldsymbol{z}}}^{{\boldsymbol{+}}}{\boldsymbol{z\text{'}}}$ where ${\boldsymbol{z\text{'}}}$ was the orthogonal matrix of latents obtained after the Gram–Schmidt procedure and ${{\boldsymbol{z}}}^{{\boldsymbol{+}}}$ is the Moore-Penrose pseudoinverse of matrix ${\boldsymbol{z}}.$ Once ${\boldsymbol{B}}$ is identified, we also transform the **Θ** matrix (${\boldsymbol{\Theta }}{\boldsymbol{\to }}{{\boldsymbol{\Theta }}}^{\prime}\,{\boldsymbol{=}}\,{{\boldsymbol{B}}}^{{\boldsymbol{-}}{\mathbf{1}}}{\boldsymbol{\Theta }}$). At the end of this procedure, we end up with orthonormal latents ${{\boldsymbol{z}}}^{{\boldsymbol{{\prime} }}}$ and corresponding features ${\boldsymbol{\Theta }}^{\prime}$ that correspond to the same abundances as ***z*** and **Θ**. For the sake of simplicity of notation, we drop the primes.

Next, we model the dynamics of the orthonormal latents using a linear dynamical system:

13 $${z}_{t+1,k}=\sum\limits _{{k}^{{\prime} }}{A}_{k{k}^{{\prime} }}{z}_{{{tk}}^{{\prime} }}+{u}_{k}+{\eta }_{k}(t)$$

where we assume that ${A}_{k{k}^{{\prime} }}={A}_{{k}^{{\prime} }k}$ and ${\eta }_{k}(t)$ are Gaussian distributed uncorrelated noise vectors: ${\langle \eta }_{k}\left({t}_{1}\right){n}_{{k}^{{\prime} }}\left({t}_{2}\right)\rangle ={\delta }_{12}{\delta }_{k{k}^{{\prime} }}$ where ${\delta }_{{ab}}$ is the Kronecker delta function. Our task is to find the symmetric interaction matrix ***A*** and the vector ***u*** that fits this model. We achieve this using squared error minimization. We write

14 $$E\left({\boldsymbol{A}}{\boldsymbol{,}}{\boldsymbol{u}}\right)=\sum\limits _{t}{\left({z}_{{tk}}-{z}_{{tk}}^{{pred}}\right)}^{2}$$

where ${z}_{{tk}}$ is the inferred latent and ${z}_{{tk}}^{{pred}}$ is the corresponding prediction using ${z}_{t-1,k}$ and Eq. (13). We restrict the summation only over time points *t* such that measurements are available for time points *t* and $t-1$. When there are no missing time points/samples, Eq. (14) can be minimized analytically. However, in real microbiome time series, samples are often missing. In that case, we propagate the latents for the missing samples using the dynamical Eq. (13). This makes the problem nonlinear as the dynamical propagation involves matrix multiplication. Therefore, to obtain a matrix ***A*** that minimizes the error in Eq. (14), we use simulated annealing. Once the matrix ***A*** is identified, we transform the orthonormal latents ${z}_{{tk}}$ into ecological normal modes ${y}_{{tk}}$ as described in the Results section.

The scripts for obtaining ECNs ***y*** and corresponding loadings **Φ** from read count data can be found at: https://github.com/mayar-shahin/EMBED.

In short, the steps involved in inferring the ECNs and the corresponding loadings are as follows.

- Start with the $T\times O\times S$ OTU-table and a chosen latent space dimension *K*. Randomly initialize the *T* × *K* matrix of latents ${\boldsymbol{z}}$ and the $K\times O\times S$ matrix of features **Θ**. In our implementation on github, we stack multiple subjects to create a $K\times (O\times S)$ matrix.
- Perform gradient ascent using Eqs. (11) and (12) to obtain the latents and the features.
- Use the Gram–Schmidt procedure to obtain an orthonormal set of latents ${\boldsymbol{z}}^{\prime}$ from the original latents ${\boldsymbol{z}}$. Obtain the $K\times K$ rotation matrix ${\boldsymbol{B}}\,{\boldsymbol{=}}\,{{\boldsymbol{z}}}^{{\boldsymbol{+}}}{\boldsymbol{z}}^{\prime}$ and transform the features ${{\boldsymbol{\Theta }}}^{\prime}\,{\boldsymbol{=}}\,{{\bf{B}}}^{{\boldsymbol{-}}{\boldsymbol{1}}}{\boldsymbol{\Theta }}$. The new orthonormal latents ${\boldsymbol{z}}^{\prime}$ and features ${{\boldsymbol{\Theta }}}^{\prime}$ fit the data to the same degree of accuracy as the original latents ${\boldsymbol{z}}$ and features ${\boldsymbol{\Theta }}$.
- Find the symmetric interaction matrix ***A*** by minimizing the squared error in Eq. (14) using simulated annealing. Diagonalize the interaction matrix ${\boldsymbol{A}}\,{\boldsymbol{=}}\,{{\boldsymbol{v}}}^{{\boldsymbol{T}}}{\boldsymbol{\Lambda }}{\boldsymbol{v}}$. Obtain the ECNs, ${{\boldsymbol{y}}}_{t}={\boldsymbol{v}}{{\boldsymbol{z}}}_{t}$ and the corresponding loadings ${\boldsymbol{\Phi }}\,{\boldsymbol{=}}\,{{\boldsymbol{v}}}^{T}{\boldsymbol{\theta }}$.

We note that in the current work, our goal was to use the dynamical model to obtain a reorientation of the latent variables, rather than fitting the latent variables to a decaying first-order dynamics. An alternative approach to simultaneously fit the dynamical model and the embedding model to the data. Specifically, we can write the total likelihood

15 $$L=\sum\limits _{t,o,s}{n}_{{os}}\left(t\right){\rm{log }}{q}_{{os}}\left(t\right)-\frac{\beta }{{2\sigma }^{2}}\sum\limits _{t,l}\left({z}_{k}\left(t+1\right)-\sum\limits _{{k}^{{\prime} }}{A}_{k{k}^{{\prime} }}{z}_{{k}^{{\prime} }}\left(t\right)-{u}_{k}\right).$$

that combines both model fit to data and the dynamics of the latent variables. In Eq. (15), we have assumed a Gaussian distribution for the noise in the linear dynamics with standard deviation $\sigma .$ We denote by *β* the hyperparameter that dictates the relative contribution of the data likelihood and the latent dynamics to the overall likelihood. Notably, *β* is a hyperparameter and is not a priori known. Therefore, our calculations can therefore be thought of as a limit where *β* is small.

### Reporting summary

Further information on research design is available in the Nature Research Reporting Summary linked to this article.

## Supplementary information

Supplementary text, figures, and tables

Reporting Summary

## Data Availability

All data and code related to the manuscript are available at https://github.com/mayar-shahin/EMBED.

## Code availability

All code related to the manuscript is available at https://github.com/mayar-shahin/EMBED.
