# Supplementary material for: EMBED: Essential MicroBiomE Dynamics, a dimensionality reduction approach for longitudinal microbiome studies
Source: NPJ Syst Biol Appl. 2023 Jun 20;9:26. doi: 10.1038/s41540-023-00285-6 (PMC10282069; doi:10.1038/s41540-023-00285-6)
Supplement: Supplementary file 1 — Supplementary text, figures, and tables [file 41540_2023_285_MOESM1_ESM.docx]

**Supplementary Information**

All our scripts are available on <https://github.com/mayar-shahin/EMBED>

**Supplementary Methods**

1. **Generating *in silico* data using the multispecies Lotka-Volterra model with competitive and cooperative interactions.**

The multispecies Lotka-Volterra model is one of the most popular models to study microbiome dynamics^6^. We use a discrete time multispecies Lotka-Volterra model introduced by Kilpatric and Ives^7^. Briefly, the abundances of species are governed by the following equation:

$$x_{o}\left( t+1 \right)=x_{o}\left( t \right)\exp\left[ r_{o}\left( 1-\frac{x_{o}\left( t \right)+\sum_{p} \alpha_{op}x_{p}(t)}{K_{o}} \right)+\epsilon_{o}(t) \right]. (S1)$$

In Eq. 16, $x_{o}\left( t \right)$ is the abundance of species $"o"$ at time $t$, $r_{o}$ is the species-specific growth rate, $K_{o}$ is the species-specific carrying capacity, $\alpha_{op}$ is the pairwise interaction matrix, and $\epsilon_{o}$ is uncorrelated Gaussian distributed noise.

For our simulations, we chose a system with $O_{total}=200$ organisms. The matrix of interactions $\boldsymbol{\alpha}$ was assumed to have both positive and negative entries reflecting competitive and cooperative interactions. The entries of the interaction matrix were chosen from a Gaussian distribution with mean zero and standard deviation 0.01. The species intrinsic growth rates were chosen from a uniform random distribution between 0 and 0.01. The carrying capacities were chosen from a uniform random distribution between 0 and 100. Finally, the standard deviation of the noise was set to 0.5. A community of $O_{total}$ organisms was initialized using random initial abundances sample uniformly between 0 and 1 and propagated for 300 timesteps. Only the last 30 timesteps were used for further analysis. The mean abundances of all species in the last 30 timesteps were calculated. The species whose mean abundances were less than 0.1% were clubbed together as a single species. This protocol typically led to an ecosystem with 50-100 species. Finally, the species abundances were normalized and then sampled using a multinomial distribution with sequencing depth of 10000. This data was used for further analysis. To obtain stable simulations using an oscillatory carrying capacity, for each OTU, we chose a random baseline carrying capacity between 100 and 200 to which we added an oscillation with a period of 3 time-steps and an amplitude of 50, with a time delay chosen uniformly between 0 and 0.5.

1. **Fitting CTF and sparse vector autoregressive models to microbiome abundance data**

**CTF:** To fit CTF to microbiome abundance data, we used the python package GEMELLI provided by Martino et al. (github link: https://github.com/biocore/gemelli). We slightly edited the script to output the individual components of the tensor factorization together with the reconstruction. These edited scripts are provided on our github (link: https://github.com/mayar-shahin/EMBED).

**Sparse autoregressive model:** Fitting first order sparse autoregressive models to relative abundance time series may lead to unnormalized model fits. To avoid this, we first converted the time series data using the CLR transform and then fit the CLR-transformed data to a first order sparse autoregressive model with L1 regularization on the parameters imposed using a Lagrange multiplier. To do so we used the scikit-learn Lasso function^8^.

We tuned the Lagrange multiplier such that the number of parameters in the autoregressive model was equal to the number of parameters in EMBED/CTF (EMBED and CTF had the same number of parameters for a given latent space dimension (# of parameters = $K\times O+K\times T$ where O is the number of organisms and T is the number of time points for a single subject time series). The fitted model was exponentiated and normalized to obtain positive valued normalized relative abundance time series. To avoid taking logarithms of zero abundances we replaced zeros with ${10}^{-6}$ and renormalized the data. The predicted values of these zero entries were reset to 0 when model predictions were analyzed.

1. **Details of the specific analyses in the manuscript**

**Figure 2g:** We tested how accurately the three methods reconstruct daily abundance change of OTUs. To that end, for each dataset, we estimated the log ratio of daily abundance change $\Delta=\log_{10} \frac{x_{o}(t+1)}{x_{o}(t)}$ across all OTUs and all days both in the data and in the reconstructed time series. Only those instances were considered when both the numerator and the denominator were non-zero in the data. We evaluated the absolute error ${\delta=|\Delta-\Delta}_{M}|$ between the observed abundance change and the reconstructed one $\Delta_{M}$ (M = EMBED/CTF/Lasso). For each dataset, each OTU, and each time point we obtained one value $\delta$ (if no zeros were involved). Next, we divided the abundances at time t in bins of 5 percentiles and combined all $\delta$ values for each bin. In panel (g), we plot the average error for each of the 5-percentile intervals (error bars represent standard errors of the mean).

**Figure 2h:** We tested the interpretation of the time-ordination (lower dimensional embedding for the time) in EMBED and CTF. This analysis could only be performed for methods that obtain time-ordination and therefore was not performed for Lasso. The re-orientation $\boldsymbol{z\to y}$ of latents using a dynamical model (Eq. 3 and 4 of main text) allows us to identify independent directions of significant collective dynamics in the microbiome without changing the accuracy of model predictions. In contrast, any other orthogonal decomposition of the microbiome time series that does not explicitly model dynamics is likely to result in a latent space description that involves a mixture of independent modes. To test the dynamical independence of ECNs, we used publicly available time series. Each time series was approximated using EMBED and CTF using $K=5$ latent space dimensions. To test whether the specificity of taxon-ECN correlations due to the accuracy of the EMBED-based reconstruction, we performed SVD on the $\boldsymbol{z\theta}$ matrix prior to the re-orientation step (Eqs. 3 and 4 of the main text) to obtain orthonormal latents $\boldsymbol{y}_{SVD}$ that *did not* consider the longitudinal nature of the data.

Next, we computed the Pearson correlation coefficient between longitudinal trajectories of individual OTUs and inferred ECNs, the corresponding CTF-based temporal components, and SVD-based orthonormal latents $\boldsymbol{y}_{SVD}$ respectively. For each analysis, we only considered OTUs whose correlation coefficients were above a 5% FDR using the Benjamini-Hochberg procedure.

For each simulated community trajectory, we obtained the fraction of OTUs that correlated with only 1 ECN, with only 1 CTF component, and with only 1 SVD component. Similarly, we also obtained fractions of OTUs that correlated with more than 1 ECN, CTF, and SVD component respectively. We then performed the Wilcoxon signed rank test on 50 such fractions obtained using the 50 simulations. The p-value are reported in Supplementary Table 1.

**Figure 2i:** In panel (i) of Figure 2, we show the precision of EMBED-based reconstruction. To that end, we generated 50 different simulations of ground truth trajectories using the multispecies Lotka-Volterra model. Using different sequencing depths, two sets of read counts were sampled using the same ground truth abundances. EMBED, CTF, and Lasso was used to model the observed read counts. We evaluated the Jensen-Shannon divergence between the two learned models. The Wilcoxon signed rank test $p$ values for comparison between EMBED, CTF, and Lasso are reported in Supplementary Table 2.

**Statistical justification of claims about trends in ECNs**

1. We claimed that the inferred ECN $y_{1}(t)$ for the antibiotics and the diet datasets were approximately constant over time. To assess the statistical significance of this statement, we compared the coefficient of variation of absolute values in $y_{1}(t)$ to coefficient of variations of absolute values of all other ECNs using the student’s t-test. For the diet dataset, the coefficient of variation of $y_{1}(t)$ was $CV_{1}=0.08$ which was significantly smaller than the CVs of other ECNs ($CV_{2-5}=0.59\pm0.09, t-test p=0.02$). Similarly, for the antibiotic dataset, the coefficient of variation of $y_{1}(t)$ was $CV_{1}=0.12$ which was significantly smaller than the CVs of other ECNs ($CV_{2-4}=0.96\pm0.13, t-test p=0.028$).
2. We claimed that ECNs $y_{2}(t)$ and $y_{3}(t)$ inferred from the diet dataset exhibited oscillatory behavior. To test this, we fit an oscillatory model to these ECNs and evaluated its root mean squared error. We next compared this root mean squared error to the root mean squared errors obtained when fitting an oscillatory model to 100 random shuffling of the data. In both cases, we found that the root mean squared error was significantly smaller for $y_{2}(t)$ and $y_{3}(t)$ compared to the corresponding shuffled data with t-test $p=0.009$ for $y_{2}(t)$ and $p=0.002$for $y_{3}(t)$ respectively.
3. We claimed that inferred ECN $y_{4}(t)$ exhibited a linear drift and $y_{5}(t)$ showed a U-shaped recovery in the diet dataset. To test the significance of these statements, we performed the analysis as above but with a linear and a quadratic fit respectively. Here too, we found that the root mean squared error was significantly smaller for $y_{4}(t)$ and $y_{5}(t)$ compared to shuffled data with t-test $p=0.01$ for $y_{4}(t)$ and $p=0.02$for $y_{5}(t)$ respectively.
4. We claimed that ECN $y_{2}(t)$ inferred from the antibiotics dataset responded to the first dose but did not respond to the second dose. In contrast, we claimed that ECN $y_{3}(t)$ only responded to the second dose but not to the first dose. To evaluate extreme departure from normal behavior, we first z-transformed the two ECNs. We observe that there are three consecutive points in $y_{2}(t)$ with z > 1 right after the first dose and the rest of the ECN is almost always between [-1, 1]. To test whether three consecutive high z-points can occur by chance before the second antibiotic dose, we calculated the probability of having three consecutive points above z > 1 before day 14 ($p=0.004$). Similarly, to test our statement about $y_{3}(t)$, we calculated the probability of having four consecutive points below z = -1 after the second dose ($p=0.0006$) (Supplementary Figure 13).

**Phylogenetic similarity between OTUs belonging to the same group:** EMBED-based inference is agnostic to phylogeny and does not impose any dynamical similarity on evolutionarily related OTUs. Yet, OTU-specific features can be used to identify OTUs exhibiting similar dynamics.

To understand whether the similarity in dynamics can be explained by similarity in phylogeny, we analyzed phylogenetic similarity indices for OTUs within the same group and for OTUs in different groups (Figures 3 and 4 in the main text). Specifically, for each OTU, we constructed a categorical vector of phylogenetic descriptors (phylum, class, order, family, and genus). Next, to assess dynamical similarity at any given level of phylogenetic resolution, we evaluated pairwise Hamming distance using the sub-vector that comprised the specified phylogenetic resolution and all coarser resolutions. For example, when comparing phylogenetic similarity at the family level, we described the OTUs using a 4-dimensional categorical vector (phylum, class, order, and family).

Next, for each OTU and for a given phylogenetic resolution, we calculated the average phylogenetic distance for all other OTUs that belonged to its dynamical group (the in-group distance) and the average phylogenetic distance for all other OTUs that did not belong to its dynamical group (the out-group distance). If the in-group distance was significantly lower than the out-group distance across all OTUs, we can conclude that there are broad phylogenetic signals in dynamics at the ecosystem level. We tested this using the rank sum test (Supplementary Table 6).

Interestingly, we found that while for the diet study, we could not detect any broad signals for phylogeny, in the antibiotics study, we found that OTUs with similar phylogeny tended to belong to the same dynamical group

1. **Details of the two investigated datasets**

**Murine gut microbiome response to oscillating diet:** We downloaded the microbiome abundance time series data on mice fed an alternating diet of high fat high sugar chow (HFHS) and low-fat plant polysaccharide chow (LFPP) from Carmody et al.^9^ as described previously^10^. Each mouse that was subjected to an oscillatory diet was treated separately. Based on our previous work on technical noise in 16S measurements, we only analyzed OTUs with mean abundances > 0.1%^4^ averaged across all time points and mice. On every day, the abundances of the rest of the OTUs were lumped together in a single meta-species.

**Murine gut microbiome response to antibiotics:** We downloaded microbiome abundance data from Ng et al.^11^. We focused on the data where mice were administered the antibiotic ciprofloxacin. Out of the 10 cages in which the mice were housed, we omitted data from cages 2, 4, 5, and 8 where many time points were missing. As above, we analyzed OTUs with mean abundance > 0.1% and combined the rest of the OTUs in a meta-species.

**Supplementary Figures**

**Supplementary** **Figure 1:** Figure 2 panels (a-c) for latent space dimension K = 3 (top) and K = 4 (bottom) for publicly available datasets

**Supplementary** **Figure 2:** Figure 2 panels (d-f) for latent space dimension K = 3 (top) and K = 4 (bottom) for GLV simulations

**Supplementary** **Figure 3:** Figure 2 panels (d-f) for read depth of 2500

**Supplementary** **Figure 4:** Figure 2 panels (d-f) for multivariate Lotka-Volterra simulations with oscillating carrying capacities. We used a latent space dimension K=5 and read depth of 10000

**Supplementary** **Figure 5:** Abundance time series of individual OTUs in the diet oscillation study. The gray lines represent abundances in individual subjects. The dark lines represent averages over subjects. The colors represent the cluster identities in main text Figure 3. HFHS: High Fat High Sugar diet and LFPP: Low Fat Plat Polysaccharide diet

**Supplementary** **Figure 6:** Scatter plot showing the accuracy of reconstruction of abundance time series for the diet and the antibiotic study

**Supplementary** **Figure 7**: Plot showing that the ecological normal modes (ECNs) inferred using EMBED are unique (up to a sign) for the two data sets considered in detail in this manuscript. The x- and the y-axis represent the ECNs inferred in two independent runs

**
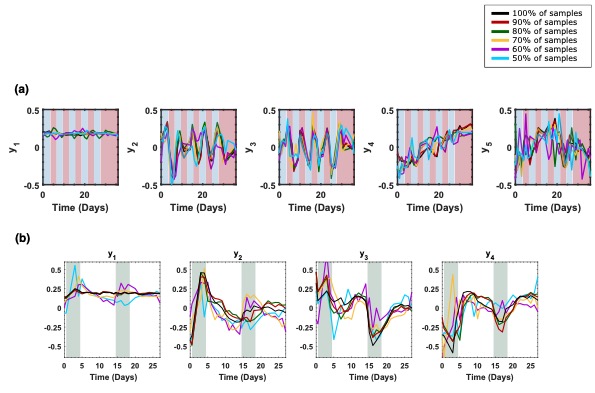
**

**Supplementary** **Figure 8:** Similarity between inferred ECNs when samples (microbiome compositions on individual days) from hosts were randomly removed. Colors represent different fractions of removed samples. Panel (a) represents the diet perturbation study and panel (b) represents the antibiotics study

**
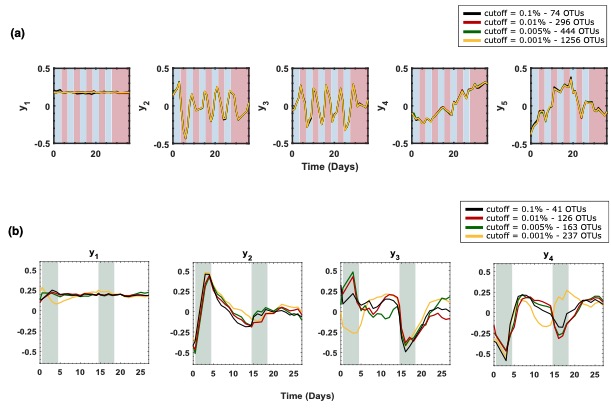
**

**Supplementary** **Figure 9:** Similarity between inferred ECNs when OTU inclusion criterion was modified to include OTUs with mean abundances ranging from 0.1% (presented in the main text) up to 0.001. Colors represent different inclusion criteria. Panel (a) represents the diet perturbation study and panel (b) represents the antibiotics study

**Supplementary** **Figure 10:** Abundance time series of individual OTUs in the antibiotics-treatment study. The gray lines represent abundances in individual subjects. The dark lines represent averages over subjects. The colors represent the cluster identities in main text Figure 4. The gray bars represent the duration of time when the antibiotic was administered

**Supplementary** **Figure 11:** Inferred ECNs for four human datasets

**Supplementary** **Figure 12:** KL divergence between reconstructed community abundances and measured community abundances, average mean squared errors of OTU-specific trajectories (averaged across OTUs), and OTU-averaged Pearson correlation coefficient between OTU-specific trajectories and the corresponding reconstruction as a function of K, the dimension of the latent space embedding


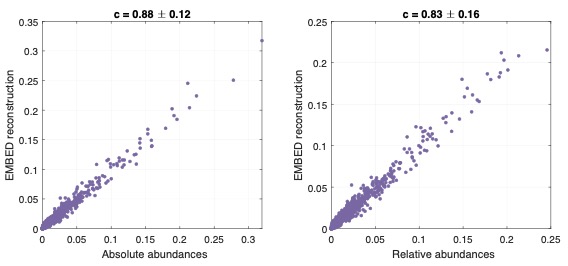


**Supplementary** **Figure 13:** Scatter plot of measured abundance (absolute abundance on the right, relative abundance on the left) vs. the corresponding EMBED-based reconstruction

**
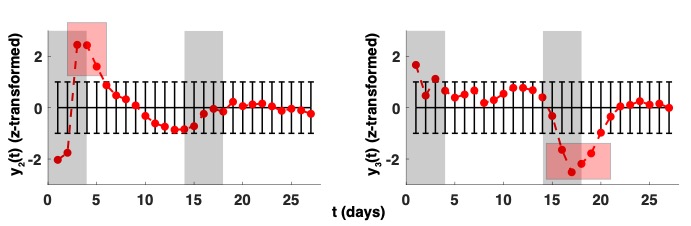
**

**Supplementary** **Figure 14:** Figure showing z-transformed ECNs $y_{2}(t)$ and $y_{3}\left( t \right)$ in the antibiotics dataset. The instances where we claimed that the ECN “responds” to the environmental perturbation (in this case, antibiotics) are shown in red squares

**Supplementary Tables**

**Supplementary Table 1.** p-values of the Wilcoxon signed rank test performed on each two different methods’ results. The test is between two methods’ lists of fractions of OTUs correlating with 1 (or more than 1 ECN) for different data sets (shown in Main Text Figure 2h)

**Supplementary Table 2**: p-values of the Wilcoxon signed rank test performed on the Jensen-Shannon divergences (symmetric Kullback-Leibler divergence) between two models learned from two different multinomial samplings of the same underlying ground truth abundances for embed and other methods (shown in Main Text Figure 2i)

**Supplementary Table 3**:The cosine similarity between ECNs inferred using all measured samples and ECNs inferred with only a percentage of samples retained in the training (90/80/70/60/50%).

**Supplementary Table 4**: The cosine similarity between inferred ECNs when the OTU inclusion criteria was relaxed to include a larger number of OTUs and the ECNs inferred using an OTU inclusion criterion of 0.1% mean relative abundance (as in Main Text Figure 3).

**Supplementary Table 5**: Phylogeny of OTUs that exhibit non-oscillatory behaviors extracted using values of $\Phi_{4}$ and $\Phi_{5}$ (shown in Main Text Figure 3c)

**Supplementary Table 6**: Number of occurrences of bacteria of the genus Oscillibacter in the top 10 OTUs with similar dynamics across all subjects (shown in Main Text Figure 3e), compared to the number of occurrences in the data set. The comparison is shown at higher taxonomical levels as well.

**Supplementary Table 7**: p-value for the Wilcoxon signed rank test performed on average in-group phylogenetic distance and average out-group phylogenetic distance over all OTUs.

**Supplementary Material References**

1. Dixit, P. D. Thermodynamic inference of data manifolds. *Phys. Rev. Res.* **2**, 023201 (2020).

2. Gloor, G. B., Wu, J. R., Pawlowsky-Glahn, V. & Egozcue, J. J. It’s all relative: analyzing microbiome data as compositions. *Ann. Epidemiol.* **26**, 322–329 (2016).

3. Stämmler, F. *et al.* Adjusting microbiome profiles for differences in microbial load by spike-in bacteria. *Microbiome* **4**, 28 (2016).

4. Ji, B. W. *et al.* Quantifying spatiotemporal variability and noise in absolute microbiota abundances using replicate sampling. *Nat. Methods* **16**, 731–736 (2019).

5. IBDMDB Investigators *et al.* Multi-omics of the gut microbial ecosystem in inflammatory bowel diseases. *Nature* **569**, 655–662 (2019).

6. Bucci, V. & Xavier, J. B. Towards Predictive Models of the Human Gut Microbiome. *J. Mol. Biol.* **426**, 3907–3916 (2014).

7. Kilpatrick, A. M. & Ives, A. R. Species interactions can explain Taylor’s power law for ecological time series. *Nature* **422**, 65–68 (2003).

8. Buitinck, L. & et al. API design for machine learning software: experiences from scikit-learn. in 108–122.

9. Carmody, R. N. *et al.* Diet Dominates Host Genotype in Shaping the Murine Gut Microbiota. *Cell Host Microbe* **17**, 72–84 (2015).

10. Ji, B. W., Sheth, R. U., Dixit, P. D., Tchourine, K. & Vitkup, D. Macroecological dynamics of gut microbiota. *Nat. Microbiol.* **5**, 768–775 (2020).

11. Ng, K. M. *et al.* Recovery of the Gut Microbiota after Antibiotics Depends on Host Diet, Community Context, and Environmental Reservoirs. *Cell Host Microbe* **26**, 650-665.e4 (2019).
